# Modulation of Ethanol-Metabolizing Enzymes by Developmental Lead Exposure: Effects in Voluntary Ethanol Consumption

**DOI:** 10.3389/fnbeh.2017.00095

**Published:** 2017-05-23

**Authors:** Miriam B. Virgolini, Mara S. Mattalloni, Paula A. Albrecht, Romina Deza-Ponzio, Liliana M. Cancela

**Affiliations:** IFEC-CONICET, Departamento de Farmacología, Facultad de Ciencias Químicas, Universidad Nacional de CórdobaCórdoba, Argentina

**Keywords:** ethanol, acetaldehyde, lead-exposure, catalase, ALDH2

## Abstract

This review article provides evidence of the impact of the environmental contaminant lead (Pb) on the pattern of the motivational effects of ethanol (EtOH). To find a mechanism that explains this interaction, the focus of this review article is on central EtOH metabolism and the participating enzymes, as key factors in the modulation of brain acetaldehyde (ACD) accumulation and resulting effect on EtOH intake. Catalase (CAT) seems a good candidate for the shared mechanism between Pb and EtOH due to both its antioxidant and its brain EtOH-metabolizing properties. CAT overactivation was reported to increase EtOH consumption, while CAT blockade reduced it, and both scenarios were modified by Pb exposure, probably as the result of elevated brain and blood CAT activity. Likewise, the motivational effects of EtOH were enhanced when brain ACD metabolism was prevented by ALDH2 inhibition, even in the Pb animals that evidenced reduced brain ALDH2 activity after chronic EtOH intake. Overall, these results suggest that brain EtOH metabolizing enzymes are modulated by Pb exposure with resultant central ACD accumulation and a prevalence of the reinforcing effects of the metabolite in brain against the aversive peripheral ACD accumulation. They also support the idea that early exposure to an environmental contaminant, even at low doses, predisposes at a later age to differential reactivity to challenging events, increasing, in this case, vulnerability to acquiring addictive behaviors, including excessive EtOH intake.

## Introduction

“The Barker hypothesis” (Osmond and Barker, 2000) first popularized the concept that parameters related to fetal, infant and childhood growth may be predictors of disease in later life. The original hypothesis has been extended to a range of components of the developmental environment such as the mother’s nutrition, stress levels, lifestyle and exposure to chemicals, all factors that may play a powerful role in influencing later susceptibility to challenging events. Based on these considerations, this review article provides behavioral and biochemical evidence that aims to support the idea that early-life exposure to lead (Pb), an environmental neurotoxicant, produces an “imprint” in CNS functionality. We propose that this experience has health consequences over the life span, increasing vulnerability to addictive behaviors, in this case to the motivational responses to ethanol (EtOH), with brain acetaldehyde (ACD) and EtOH metabolizing enzymes playing a crucial role.

## Lead, Ethanol and the Two Faces of Reinforcement

Although a non-essential metal and with widely restricted industrial uses, Pb is present in the environment and in living organisms. Alarmingly, early-life Pb exposure even in trace amounts induces neurobehavioral manifestations that may not be evident until later in life (Vorvolakos et al., 2016). They include hyperactivity, cognitive deficits and altered responses to drugs of abuse including EtOH.

From the clinical perspective, a relationship between Pb and EtOH has been described (Cezard et al., 1992). Animals chronically exposed to high Pb levels during adulthood, showed higher, although less efficient, lever pressing for EtOH in a self-administration test, associated with increased EtOH intake (Nation et al., 1987). More recently, it was shown EtOH-induced hyperlocomotion after acute Pb administration (Correa et al., 2001). Similarly, perinatal low-level Pb exposure enhanced EtOH intake in a daily 2-h EtOH/water free-choice sessions. Moreover, Pb-exposed animals also showed elevated response rates in a FR-2 schedule of behavior associated with a higher breaking point compared to controls, evidencing their motivation to self-administer EtOH (Mattalloni et al., 2013).

Thus, to find a mechanism that explains these differential effects, the concepts of positive and negative reinforcing must be introduced. It is known that drug addiction is a process that progresses from an early condition of positive reinforcement, evidenced by the euphorizing and stimulant effects of the drug (compulsive desire for pleasure), to a later state of negative reinforcement, evidenced as dysphoria and anxiety as a result of drug removal (compulsive desire for relief). Thus, the two main sources of reinforcement play key roles in the allostatic processes that lead to drug abuse (Wise and Koob, 2014). Both aspects will be mentioned in this review article, with a focus on the positive reinforcement perspective, particularly related to EtOH and its bioproducts, with developmental Pb exposure as a determinant factor in the vulnerability of these animals to the motivational effects of EtOH.

A putative explanation for the Pb/EtOH interaction supported by the *negative reinforcement perspective* is based on the tension-reduction hypothesis (Pohorecky, 1990). This proposes that the anxiolytic properties of EtOH are the main factors that lead some individuals to consume excessive amounts of the drug to relieve negative emotionality. This mechanism involves both the hypothalamic-pituitary adrenal axis with corticosterone secretion as the final output (Fahlke et al., 1994), as well as the extrahypothalamic systems including the extended amygdala (Koob, 2008). The increased susceptibility to EtOH-anxiolytic effects and the enhanced EtOH intake reported in perinatally Pb-exposed animals was associated with elevated basal corticosterone levels (Virgolini et al., 1999). Thus, it is proposed that Pb-treated animals would ingest EtOH to diminish their basal anxiety in an attempt to cope with stressful situations. This line of research was not further investigated and deserves future endeavors.

The *positive reinforcement view*, on the other hand, is related to the motivational and stimulant effects of EtOH, mediated through the reported ability of EtOH, ACD and salsolinol (a tetrahydroisoquinoline product of dopamine (DA) and ACD condensation) to facilitate DA neurotransmission in the mesolimbic circuit. Moreover, central administration of EtOH (or its bioproducts) induces hyperlocomotion, conditioned place preference and promotes EtOH intake (reviewed in: Quertemont et al., 2005; Correa et al., 2012; Hipólito et al., 2012; Deehan et al., 2013; Israel et al., 2015; Peana et al., 2016). Therefore, the present review will particularly emphasize the modulation that the environmental neurotoxicant Pb exerts on the enzymes involved in central EtOH metabolism, given the positive reinforcing properties of EtOH, ACD and salsolinol.

With the reported low ADH activity in the brain, the CAT-H_2_O_2_ system in addition to being a peroxisomal redox regulator is the key enzyme involved in H_2_O_2_-dependent brain EtOH oxidation to ACD (Vetrano et al., 2005). It should be mentioned that blood catalase (CAT) activity is positively correlated with EtOH consumption in both rats (Amit and Aragon, 1988) and humans (Koechling and Amit, 1992). Several reports indicate that CAT activity is decreased after chronic adult Pb exposure in the brain (Jindal and Gill, 1999), liver (Flora et al., 2012a) and blood (Sajitha et al., 2010). Interestingly, developmental exposure to high Pb doses is able to increase CAT activity (brain: Valenzuela et al., 1989; brain, liver and heart: Somashekaraiah et al., 1992). Moreover, cumulative evidence demonstrated that acute (but not chronic) Pb administration raises brain CAT levels and increases the locomotor response to EtOH in mice (Correa et al., 1999, 2001). Similarly, we have reported that developmental Pb exposure increased basal blood CAT activity in periadolescent rats, an effect that persisted throughout their lifetime and was potentiated by EtOH intake. Although no differences between groups were observed in whole brain CAT activity, there was a region-specific increase in the Pb-exposed hippocampus and cerebellum, indicating that CAT-mediated EtOH oxidation is not homogeneous throughout the brain (Mattalloni et al., 2013, 2017).

On the other hand, ACD removal is mediated by ALDH2, a mitochondrial enzyme that belongs to the ALDH superfamily and catalyzes both brain and liver ACD oxidation to acetic acid (Crabb et al., 2004). The two evidences of an interaction between Pb and ALDH2 have shown that adult Pb exposure reduced liver ALDH2 (Flora and Tandon, 1987) whereas developmental Pb-exposure reduced brain ALDH2 activity (Mattalloni et al., 2017) after chronic EtOH consumption.

Thus, based on the premise that early-life Pb exposure will interfere with EtOH metabolism, brain ACD may be noted as the common site of action of the two neurotoxicants. Pharmacological manipulations of EtOH-metabolizing enzymes attempting to modulate brain ACD accumulation will therefore be described below, with the resultant changes evidenced at both behavioral and biochemical levels.

## Pharmacological Interference of Ethanol Metabolism

**Figure 1:**
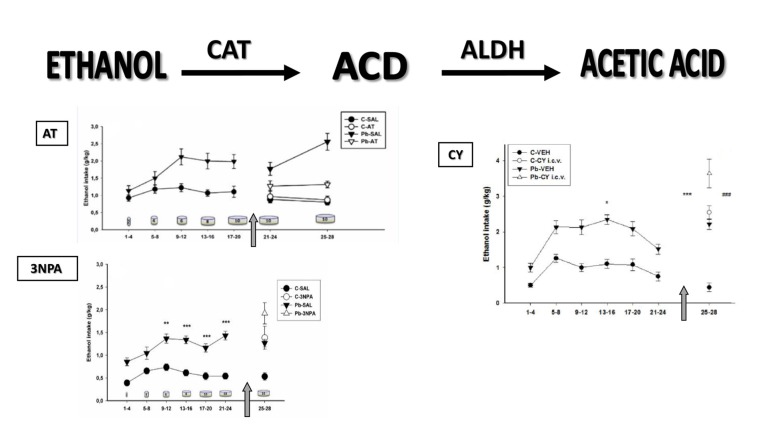
**Voluntary ethanol (EtOH) consumption measured in Wistar rats.** Data (mean expressed as grams of EtOH per kilogram of body weight ± SE) grouped in 4-day blocks along the horizontal axis (in days) that correspond to EtOH intake in response to increasing EtOH concentrations symbolized as cylinders (days 1–4: 2%; days 5–8: 4%; days 9–12: 6%; days 13–16: 8%; and days 17–28: 10%). C, control; Pb, lead; CAT, catalase; SAL, saline; VEH, vehicle. Top, left: EtOH intake in response to 3-amino 1,2,4-triazole (AT) administration. The arrow signifies the start of SAL or AT administration (days 21–24 and 25–28; 250 mg/kg i.p.). C-SAL = 10; C-AT = 11; Pb-SAL = 11; Pb-AT = 9 animals per group (Mattalloni et al., 2013). Bottom, left: EtOH intake in response to 3-nitropropionic acid (3NPA) administration. The arrow signifies the start of SAL or 3NPA administration (days 25–28; 20 mg/kg s.c.). Baseline: *denotes statistical difference compared to controls at **p < 0.01 and ***p < 0.001. C-SAL = 8; C-3NPA = 11; Pb-SAL = 9; Pb-3NPA = 9 animals per group (Mattalloni et al., 2013). Right: EtOH intake in response to intracerebroventricular cyanamide (CY) administration. The arrow signifies the start of VEH or CY administration (days 25–28; 0.3 mg i.c.v.). Baseline: *denotes differences compared to controls at *p < 0.05. CY administration: *denotes differences between the C and Pb-exposed animals injected with VEH at ***p < 0.001; ^#^denotes differences between the VEH and corresponding CY groups for both C and Pb-exposed animals at ^###^*p* < 0.001. C-VEH = 11; C-CY i.c.v.= 14; Pb-VEH = 8; Pb-CY i.c.v.= 8 animals per group (Mattalloni et al., 2017).

The next section follows the two-dimensional model of alcohol consumption hypothesized over 30 years ago, supporting the idea that “both brain CAT and ALDH may represent a biological marker system underlying the affinity of the animals to consume ethanol” (Aragon and Amit, 1985). Evidence is provided that Pb induces dynamic changes in the two main enzymes involved in brain EtOH metabolism, which may account for differential EtOH intake in response to pharmacological manipulations in these animals (Figure 1).

### Brain Acetaldehyde Formation

One of the most commonly used CAT blockers employed to modulate stimulant responses to EtOH is 3-amino 1,2,4-triazole (AT), a fungicide that produces irreversible inhibition of the CAT-H_2_O_2_ site, thereby preventing *in vivo* brain EtOH oxidation to ACD (Aragon et al., 1989). Interestingly, the only report of an interaction among Pb, CAT, AT and EtOH showed that AT was able to reverse the increase in EtOH-induced hyperlocomotion and brain CAT activity observed after acute Pb administration (Correa et al., 2001). Similarly, we have reported that AT pretreatment prevented both elevated EtOH intake and blood and brain (hippocampus and cerebellum) CAT activity in developmentally Pb-exposed animals (Mattalloni et al., 2013). The absence of these effects in the control group suggests that the enzyme inhibition requires either high H_2_O_2_ (and ROS) levels that are increased as a result of Pb exposure (Flora et al., 2012b) or the excessive EtOH intake evidenced in Pb-exposed animals.

On the other hand, CAT overactivation can be achieved by the administration of 3-nitropropionic acid (3NPA), a mycotoxin that produces an irreversible inhibition of the succinate dehydrogenase (SDH) enzyme, along with ROS elevation and increased CAT activity, with resultant EtOH-induced hyperlocomotion (Manrique et al., 2006). Thus, 3NPA induced-CAT elevation was able to increase EtOH consumption in both, the Pb-exposed and the control animals, accompanied by higher blood and brain (striatal) CAT activity in the Pb group (Mattalloni et al., 2013).

### Brain Acetaldehyde Removal

Cyanamide (CY) is a drug prescribed in some countries as a deterrent for alcoholics due to its ability to increase peripheral (aversive) ACD as a result of ALDH inhibition (Koppaka et al., 2012). Central CY administration enhanced EtOH intake in rats that had never consumed EtOH, an effect highly dependent on the CY dose (Critcher and Myers, 1987). We have demonstrated that i.c.v. CY administration inhibited brain ALDH2 and increased EtOH intake in control animals, whereas the Pb-exposed group also showed elevated EtOH intake although in the absence of brain ALDH2 inhibition (Mattalloni et al., 2017). This finding may be related to the reduced basal brain ALDH2 activity present in the Pb-exposed group, or to the fact that CY is a prodrug that, to convert itself to the active metabolite requires CAT and H_2_O_2_, a system that is modified by Pb-exposure.

## Conclusion

**Figure 2:**
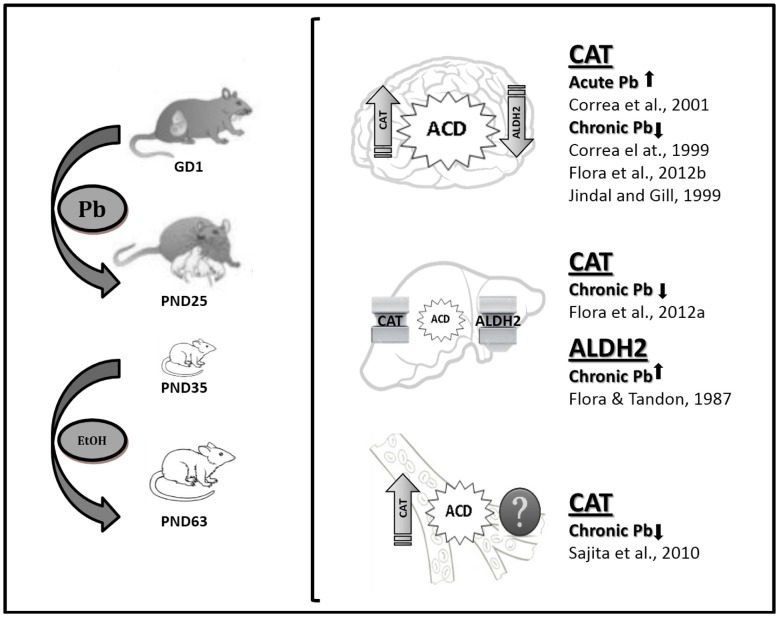
**Lead (Pb) exposure and EtOH intake with emphasis in EtOH metabolizing enzymes status.** The square bracket comprises pictures for brain, liver and blood CAT and ALDH2 status and putative acetaldehyde (ACD) accumulation in the experimental model described in Mattalloni et al. (2013, 2017; as shown on the left). The references point-out CAT and ALDH2 data reported elsewhere as result of adult acute or chronic Pb exposure in animals with chronic EtOH intake. GD, gestational day; PND, postnatal day.

This review article provides evidence of Pb modulation on the enzymes involved in either the production or the removal of brain ACD, i.e., CAT and ALDH2, the activities of which have been proposed as trait biomarkers of excessive EtOH intake (Aragon and Amit, 1985). The data demonstrate differential CAT and ALDH2 functionality in the developmentally Pb-exposed animals, with high blood and brain CAT activity and low brain ALDH2 activity, thereby promoting central ACD accumulation (Figure 2). This effect would directly influence EtOH self-administration, with Pb exposure representing a crucial variable in the behavioral and biochemical outputs described here. It can thus be postulated that one of the shared mechanisms between Pb and EtOH could be the result of differential EtOH metabolism in brain areas related to reward. Possibly, an imbalance towards a prevalence of the reinforcing effects of brain ACD vs. aversive peripheral ACD accumulation may play a key role in the differential motivational response to EtOH evidenced in Pb-exposed animals. Moreover, immunohistochemical studies have demonstrated that ALDH2 is widely expressed in the brain, with low activity in the aminergic neurons, which coincidentally are the richest in CAT expression (Zimatkin, 1991; Zimatkin and Lindros, 1996), a fact that promotes brain ACD accumulation in the mesolimbic circuit, site of the reinforcing properties of addictive drugs. Immunostaining experiments are desirable for brain CAT and ALDH2 expression in the Pb-exposed animals.

Interestingly, there are differences in EtOH metabolism over the lifetime. CAT-H_2_O_2_ system activity is higher in pups than in adults (Hamby-Mason et al., 1997), thus promoting central ACD accumulation. Brain ALDH2 activity also increases gradually, reaching the activity specific for mature animals by periadolescence (Zamatkin and Lis, 1990). Hence, Pb exposure during development may have affected the functionality of these enzymes at a time of high ACD accumulation. This assumption has important clinical implications provided that the neurobehavioral outcomes showed no evidence of a safe threshold for Pb exposure in immature organisms and the ubiquity of this environmental neurotoxicant. Thus, these results indicate the existence of prenatal programming as a consequence of early Pb exposure, an experience that would leave an imprint that later in life may be responsible for differential responsiveness to events that generate a conflict in the individual, such as the initiation in addictive behaviors.

## Author Contributions

LMC and MBV conceived and designed the experiments. MSM, RD-P and PAA performed the experiments and analyzed the data. MBV wrote and LMC contributed to the writing of the manuscript.

## Conflict of Interest Statement

The authors declare that the research was conducted in the absence of any commercial or financial relationships that could be construed as a potential conflict of interest.
